# FAO/WHO Joint Expert Meeting on Microbiological Risk Assessment (JEMRA): Twenty Years of International Microbiological Risk Assessment

**DOI:** 10.3390/foods10081873

**Published:** 2021-08-13

**Authors:** Jeffrey T. LeJeune, Kang Zhou, Christine Kopko, Haruka Igarashi

**Affiliations:** 1 Food Systems and Food Safety Division, Food and Agriculture Organization of the United Nations (FAO), Viale delle Terme di Caracalla, 00153 Rome, Italy; kang.zhou@fao.org (K.Z.); christine.kopko@fao.org (C.K.); 2 Department of Nutrition and Food Safety, World Health Organization, 20 Avenue Appia, 1211 Geneva 27, Switzerland; igarashih@who.int

**Keywords:** food safety, food microbiology, microbiological risk assessment, JEMRA

## Abstract

Since the late 1990s, the Food and Agriculture Organization (FAO) of the United Nations and the World Health Organization (WHO) has convened expert meetings and consultations to address the microbiological risk assessment (MRA). These meetings are held to provide scientific advice in response to requests for from Codex Alimentarius, the international food standard-setting body. Individuals participate in the FAO/WHO joint expert meetings on the microbiological risk assessment (JEMRA) in their personal capacity, as technical experts, yet bring diverse regional and national perspectives that contribute to practical applications, particularly for low- and middle-income countries (LMICs). Over 370 experts from around the globe have contributed to the meeting outcomes that have been published in nearly 40 monographs in the FAO/WHO microbial risk assessment (MRA) series, addressing particular food commodities with microbial hazard(s) combinations or a methodological aspect of microbial risk assessment. FAO/WHO MRA series inform Codex decision-making for the development of international standards for safe food and faire trade in food products; are consulted by risk managers such as food safety authorities and food business operators to make science-based decisions; and are used by academics to advance food safety research and educate the next generation of food safety professionals.

## 1. Introduction

The concepts of risk and risk assessment are not new. The genesis of modern risk analysis, and, in particular, risk assessment, has a long and interesting history building over centuries on mathematical and statistical principles. In his book, *Against the Gods: The Remarkable Story of Risk*, economist and historian Peter Bernstein [1] posited that risk “is a choice, rather than a fate. The actions we dare to take, which depend on how free we are to make choices, are what the story of risk is all about”. Indeed, risk assessments of various complexities have been employed to inform decision-making in gaming (gambling), economics and finance, environment, health, and a number of other areas of public policy. By the late 1990s there was growing interest in the potential of this approach to provide a transparent and reproducible pathway for the management of food safety risks. However, the freedom or ability to make choices related to food safety risks is not equally available to all.

The risk assessment process allows for the characterization of food safety risks based on the identification and characterization of hazards, particularly microbiological and chemical hazards, and the likelihood of exposure. Notably, the prevalence of foodborne pathogens, their population structure and level of virulence, in food systems may vary greatly from one food environment to another. Moreover, the likelihood of exposure to foodborne pathogens among the population is determined by steps taken to control contamination, which in itself is dependent upon stakeholders’ knowledge and skills; their desire to intervene or prevent exposure; and the enabling (opportunities) or disenabling (barriers) environment present. Thus, there is no one-size-fits-all solution to enhancing food safety. However, using systematic approaches to characterize risks and evaluate risk mitigation strategies provides a pathway to prioritize hazards and answer specific questions about risk reduction under diverse scenarios or situations.

The Codex Alimentarius Commission (CAC), established in 1963 under the Joint FAO/WHO Food Standards Programme, is the international food standards setting body with the goal of protecting consumers’ health through a safe and secure food supply and ensuring fair practices in food trade. When developing Codex texts (i.e., standards, guidelines, code of practice, and other recommendations), the CAC apply risk analysis and rely on the independent scientific advice provided by expert bodies organized by FAO/WHO.

With respect to microbiological aspects of food safety, a joint FAO/WHO expert consultation on the application of risk analysis to food standards issues was held in 1995 [2] and would represent the first in a series of meetings that would eventually lead to the formation of the FAO/WHO joint expert meeting on microbiological risk assessment (JEMRA). That consultation delineated the basic terminology and principles of risk assessment and concluded that the analysis of risks associated with microbiological hazards in foods presented unique challenges. At its 22nd session in 1997, the CAC requested FAO and WHO to convene an international advisory body specifically on the microbiological aspects of food safety to address, in particular, microbiological risk assessments [3].

In response to this request, and as follow-up on their previous activities in the area of risk analysis, FAO and WHO convened an expert consultation in 1999 to examine the issue and develop a strategy for microbiological risk assessment (MRA) in an international forum [4]. Subsequently, at its 32nd session in November 1999, the Codex Committee on Food Hygiene (CCFH) recognized that there are significant public health problems related to microbiological hazards in foods [5]. CCFH initially identified 21 pathogen-commodity combinations of concern and prioritized these according to such criteria as the significance of the public health problem, the extent of the problem in relation to geographic distribution and international trade, and the availability of data and other information with which to conduct a risk assessment [5]. That same month, CAC requested that FAO and WHO convene ad hoc expert consultations to provide advice on MRA, and recommended that these consultations be conducted according to the format previously outlined [6,7]. This resolution was subsequently approved and JEMRA was born in the year 2000.

## 2. JEMRA Scope

The work of JEMRA is defined by the aforementioned documents and is undertaken under the framework of the FAO/WHO Framework for the Provision of Scientific Advice on Food Safety and Nutrition, similar to that of the FAO/WHO joint expert committee on food additives (JECFA), the FAO/WHO joint expert meetings on pesticide residues (JMPR), the FAO/WHO joint expert meetings on nutrition (JEMNU), and other ad hoc expert meetings [8,9]. In brief, the purpose of JEMRA is to provide scientific advice to (1) CAC, primarily CCFH, to assist them in the development of standards, guidelines, and recommendations for food in international trade; and (2) FAO and WHO members to assist them in overcoming problems related to the microbiological hazards in foods and achieve a greater level of consumer protection. In this context, scientific advice is defined as, “the conclusion of a skilled evaluation taking account of the scientific evidence, including uncertainties” [9]. To achieve this goal, JEMRA activities can be clustered into two primary categories:

### 2.1. Synthesis of Scientific Information and Development of Risk Assessments

One of the main aims of JEMRA is to provide a transparent review of scientific advice on the state of the art of MRA, and to develop the means of achieving sound risk assessments of specific pathogen–commodity combinations. The work includes establishment of new risk assessment; evaluation of existing risk assessments; review of the available data and current risk assessment methodologies, highlighting their strengths and weaknesses and how they may be applied; provision of examples; and identification of ongoing data and information needs. This work often includes an evaluation of the impacts of different risk management options in the reduction or control of specific microbiological risks in food based on an analysis of the available scientific knowledge and on scientific judgement.

### 2.2. Information and Technology Transfer

Although risk managers are one of the ultimate users of the outputs from the risk assessments, this information is valuable to a broad audience of stakeholders including risk managers, risk assessors, scientists, educators, civil society, and others, especially those in low- and middle-income countries (LMICs). The documentation relating to these risk assessments is primarily communicated through the reports of FAO/WHO microbiological risk assessment series. In addition, the elaboration of guidance, guideline documents, and participation in meetings, webinars, and related media pathways are methods used by JEMRA to further assist risk managers, and others, in understanding the risk assessment process and promoting the optimal use of these tools. Many of these communication modalities serve as approachable references to those individuals who are themselves not risk assessment experts. Notably, in communicating information, it is important to convey the level of uncertainty in the current state of knowledge and in the adequacy of the available data used.

## 3. JEMRA Framework

**Figure 1:**
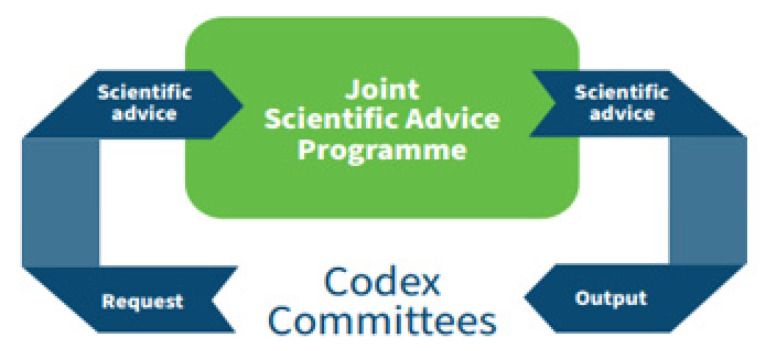
Information flow between JEMRA and the Codex Committee on Food Hygiene (CCFH).

The primary purpose of the establishment of JEMRA was to provide scientific advice to Codex. Based on their needs for information, Codex committees, primarily the CCFH, prepare specific request for information and communicate their needs for scientific advice to the JEMRA secretariat. As with other FAO/WHO Joint scientific Advice Programmes, the JEMRA secretariat works in close collaboration with Codex Committees’ group Chairs to ensure that the scientific advice of JEMRA is truly tailored to the Committee’s needs (Figure 1). Once complete, JEMRA reports back the Committees that have requested the information.

In addition to responding to requests from Codex, JEMRA may take on important risk assessment activities identified through other pathways. For example, JEMRA has addressed questions raised by their United Nations partner, the World Food Programme (WFP). JEMRA may undertake a review of past MRAs if changes in science dictate a need for an update or action is required. New and emerging trends in food safety or foodborne disease are also considered, which may be identified by FAO and WHO foresight and horizon scanning activities.

## 4. Data

FAO and WHO have established procedures for the collection of data and the selection of experts involved in the provision of scientific advice [9]. Public calls for data and experts are issued to solicit up-to-date data from all regions of the globe for JEMRA meetings. This Call is disseminated by joint secretariats as early and as broadly as possible via the Internet, Codex e-mail lists, industry, and professional associations, etc.

Quality data are the basis for sound scientific advice. Publicly available, peer-reviewed scientific literature forms the foundation of JEMRA scientific advice. In addition, it is recognized that valuable supplementary data on particular topics may exist in the grey literature, such as in government documents, reports and databases, as well as from other sources. This is particularly important and relevant when research reports are sparse from particular geographic regions. In cases where the quality of data is less than optimal, but no other data is available, the available data are taking into account, and the limitations and the uncertainty associated with it are clearly described in the final report.

## 5. JEMRA Meetings

**Figure 2:**
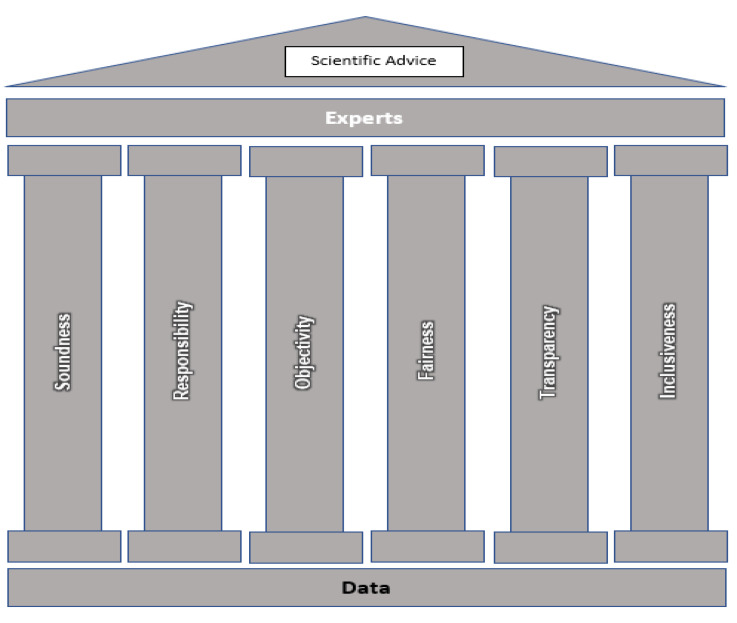
Representation of the core principles underpinning the JEMRA scientific advice framework.

JEMRA advice is generated through a process of scientific consensus obtained from expert elicitation of scientific opinion supported by data. Meetings are convened by the Joint FAO/WHO secretariats, comprising of professional staff members from FAO and WHO, who are responsible for the preparation, organization, and follow-up. The framework of JEMRA is based upon two fundamental precepts and six core principles as means to establish the credibility of the advice produced (Figure 2).

First, JEMRA achieved international recognition in the field of MRA because of its prerequisite for data and independence. These principles provide a fundamental foundation for the role of FAO and WHO as a neutral, international forum for the provision of sound scientific advice. Moreover, the advice provided, and the experts who participate are held to the following principles: (1) soundness (scientific excellence); (2) responsibility and (accountability), with respect to safeguarding the integrity of the process and holding experts answerable for their views; (3) objectivity an neutrality; (4) fairness applies to the conduct of the scientific advice process, and requires respect of all participants for each other and for their scientific views; (5) transparency, in the process whereby advice is formulated and crafted in a way that is understandable to others; and (6) inclusiveness, incorporating a balance of skills and expertise necessary for the assessment, having broad geographic input, gender diversity and inclusion, and consideration of minority scientific opinion.

Decisions on interpretation of key data, on evaluations and final conclusions are made by consensus. The scientific questions are not submitted to votes. However, in the event that no consensus can be reached a minority opinion could be expressed and recorded in the meeting report and the minority view published as annex, stating the reasons for the divergent opinion. The latter, lack of consensus, has yet to occur in the 20-year history of JEMRA.

### 5.1. Selection of Participants

All experts are invited and appointed to participate in JEMRA in their independent (personal) capacity and do not represent, their government, nor a particular industry, organization, commodity, or employer. Calls for experts are issued periodically to populate a JEMRA roster or to meet the needs of a specific upcoming meeting. As per the calls for data, calls for experts are disseminated widely and describe the background, objective and agenda of the meeting or consultation, and specify the selection criteria and process.

All applications are reviewed by a selection panel composed of representatives of FAO and WHO and at least one external expert on the relevant subject. Experts are selected based on pre-established criteria, as well as the meeting agenda, particular expertise required, geographical and gender representation and coverage of different schools of thought on the topic to be addressed. Once approved, appointees are informed, and rosters are posted on the FAO and WHO web sites [10].

The “Experts” responsibilities are to consider the questions posed, review available data, prepare draft evaluations in advance for discussion, draw appropriate conclusions, draft sections of the report and adopt the final report. A chairperson, selected among the experts, manages the meeting and other meeting participants serve as rapporteurs to document the discussions.

Usually, JEMRA meetings are held at either the FAO or WHO headquarters in Rome and Geneva, respectively. Recently, during periods of COVID-19 travel restrictions, meeting have been held virtually as an alternative to physical meetings. Such on-line meetings, although successful, present challenges for the secretariat and participants as it is difficult to schedule periods of uninterrupted work (one week for physical meeting, but three to four weeks with three to two hours per day for virtual meeting) for participants to focus uniquely on the topic a hand, when they remain in their regular work environment. Moreover, given the various time-zones represented, even shorter meeting will run at very late and very early hours for some participants. The free and casual discussion that occur during shared meals and social activities is also lost, along with new and innovative ideas that often emerge from these interactions.

In addition to the secretariat and experts, other individuals are occasionally invited to participate in JEMRA meetings when involved in subject related activities, such as representatives of international organizations; the CAC, its secretariat or one of its committees; data providers (e.g., when meetings evaluate proprietary data); etc. Such individuals are invited on the basis of their organizational affiliation rather than in their individual capacity. Their participation of these individuals, or resource persons, is usually limited to certain aspects of the meeting and they are excluded from the final decision- making process.

### 5.2. Declaration of Interests, Confidentiality Undertaking, Code of Conduct

To ensure the objectivity and independence of the scientific advice developed, all experts involved in meetings are required to declare any interests that could constitute a real, potential, or apparent conflict of interest with respect to his/her involvement.

Interests declared by experts are evaluated by the joint secretariats based on pre-defined criteria. Depending upon the nature of the (perceived) conflicts, and their potential to influence the participants judgment, individuals may be dismissed from the meeting, or participate in only portions of the meeting as a resource person. All identified real and perceived conflicts of interest and the agreement on how they are managed are disclosed at the beginning of the meeting and recorded in the meeting report. The confidentiality undertaking aims to keep the relevant information as confidential and to disclose it only to persons who are bound by similar obligations of confidentiality and non-use as are contained in the meeting.

## 6. Meeting Reports

Scientific advice generated by JEMRA is documented in technical reports published as part of the MRA series established in 2002. The experts agree upon the report content before the close of the meeting and will not backfill afterwards without the consensus from all the experts. The meeting report is then drafted by some or all of the experts, based on the available scientific evidence and the discussion during the meeting, and it is fully reviewed by all experts prior to publication. In some situations, an additional peer- or public-review process may be warranted. Meeting reports go through a thorough editorial process and FAO and WHO internal clearances, which may take several months. With the exception of editorial revisions (to reflect FAO and WHO editorial guidelines), joint secretariats do not modify or amend the interpretation of data, conclusion, recommendations or advice produced by the Experts during the meeting. To enable the findings to be disseminated more quickly, summary reports are published on the FAO and WHO web sites within a few weeks after the meeting. MRA series entails all monographs resulting from JEMRA and is available on FAO and WHO web sites.

## 7. Resources for the Provision of Scientific Advice

Travel and accommodation costs associated with experts’ participation are covered by FAO and/or WHO, but experts are not typically compensated for their time at the meeting nor for the many hours of work associated with meeting preparation and follow-up. Occasionally, financial support has been made available to experts requested to prepare in-depth background documents prior to a meeting. Thus, the quantity and quality of work performed by JEMRA relies largely on voluntary contributions provided by individual participants and the willingness of their employers to allow them to devote significant time to such work. It is important to note that this constitutes a significant in-kind contribution, which is invaluable and without which the FAO/WHO Joint Scientific Advice Programme would not exist. Joint secretariats acknowledge these contributions in communications to individual experts and their employer and experts are encouraged to include their contributions to these works as part of their professional dossiers.

FAO and WHO activities related to the provision of scientific advice on food safety and nutrition are financed by both organizations separately, using the regular programme budget resources, specified voluntary contributions as well as extra-budgetary funds from various donors. In addition, several FAO and WHO members have supported JEMRA through the secondment of officers dedicated to work on this programme.

## 8. JEMRA Outputs

Although acting in their independent capacity for these meetings, over 370 individuals with experience in academia, consumer and industry associations, government, and the private sector have participated in JEMRA over the last 20 years, many included as part of the dynamic JEMRA roster of experts [10]. Experts have been recruited from nearly 60 different countries, representing all FAO and WHO regions.

Scientific advice has taken on many different forms; from a response to a specific question, or provision of scientific information related to particular needs, to a full quantitative risk assessment. Depending on the degree of uncertainty, the output could range from a clear conclusion on risk to a recommendation to obtain additional data. The outcomes, conclusions, and recommendations of these meeting have been reported in the MRA series publications (Table 1) which provide expert scientific advice for a number of microbiological hazards and food commodity combinations (Table 2) of greatest public health concern.

In the early 2000s, regular meetings of the Joint FAO/WHO expert body to address MRA issues began. With framework documents on methodological aspects of risk assessment complete, the first tasks were directed towards identified priorities of *Listeria* spp. in ready-to-eat (RTE) foods (see MRA4 and MRA5, Table 1). This was a huge undertaking and took several years to address due to the broad scope. In the end, there was a focus on a few food products as representative of categories of food that allowed growth of *Listeria* spp. since most illnesses were linked to higher exposure doses. An expert report on the subject was first published in 2004 [11] while CCFH was also working on their guidelines with the support of this report and transformed this scientific advice into practical guidelines for *Listeria* control in RTE foods in 2008 and 2009 (Table 3). Other examples of how JEMRA scientific advice have been used by Codex are also included in Table 3.

Other examples of early topics addressed through the MRA series publications included *Cronobacter (Enterobacter) sakazakii*, in infant formula and *Salmonella* spp. in chicken and eggs. Strong focus in this former risk assessment was the emphasis on consumer behavior. A risk assessment tool was developed that allowed the risk of different preparation practices to be assessed. This led to the development of risk-based guidance for preparation of powdered infant formulae in a number of countries. Another specific success of this work was the development of a new dose response model for *Salmonella* which has been extensively used in risk assessments around the world since, with minor if any changes. Interest grew on the assessment of effectiveness of interventions to reduce and eliminate risks. The topics/pathogens investigated also grew and continues to be directed by international, regional, and national-level needs and request from CCFH.

## 9. Reflections and Future Considerations

**Figure 3:**
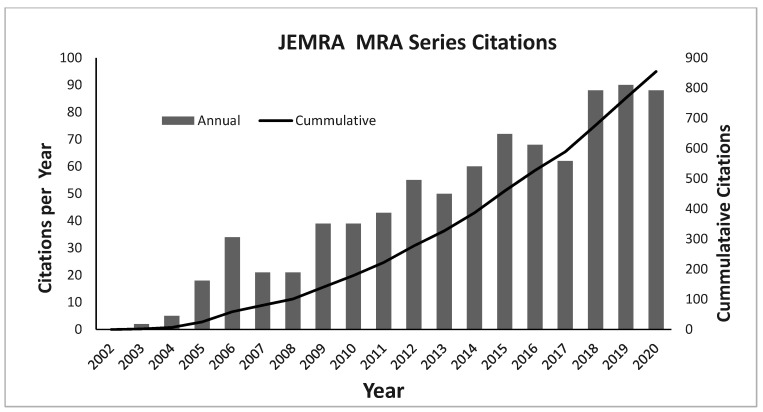
Citations of JMRA MRA publications as indexed by Web of Science (retrieved 15 June 2021).

The hallmark of these works is their scientific foundation, and over time, the scientific foundation has remained a key point, but methodologies evolved to keep pace with state-of-the-art scientific approaches and to optimally reflect what could most usefully be done at global level. In addition to their use by Codex, JEMRA documents are used for a variety of other purposes, including the use as teaching aids, justification for food safety funding request and to fuel additional research (Figure 3). Another key characteristic of the JEMRA is its global scope, with emphasis on applicability to LMICs. Undertaking this work with global scope results in the provision of valuable information on particular pathogen–commodity combination for use by risk managers at both the national, regional, and international levels. On one hand, an international approach to MRA does provide several advantages: First, information, frameworks, and tools applicable to MRA from around the world can be collected or elaborated and centralized. This facilitates both the distribution and accessibility of the knowledge, technology, and related information. Secondly, it permits the identification of areas which are similar or common to a particular region or even to all countries and it provides a means of addressing issues of international concern or issues of concern to a large number of countries. Finally, international MRA enables the identification of available data on a global scale and equally important the areas where knowledge and data are lacking. Such benefits can be further enhanced when a one health approach is used to evaluate risk and mitigation strategies with respect to broader goals of sustainable development including protecting biodiversity, reducing food loss and waste, sustainable agriculture practices, impacts of antimicrobial resistance, and the effects of climate change, to name a few.

It is, however, important to recognize that risk assessment at the international level is substantially different from risk assessment at the national level. It cannot consider the situation in all countries and therefore tends to be more generic in nature and cannot capture local scenarios and country-to-country variations, e.g., in processing, farming practices, contamination levels, consumer behavior, consumption, etc. It cannot produce a globally applicable risk estimate, i.e., one risk estimate that is valid for all countries. Due to the variation that exists such a metric would be meaningless. There are also limitations to what can be undertaken at the national level due to the availability of resources. Ultimately, international work is very dependent on national and regional expertise and data. Additional data on food safety in LMICs remains a critical gap to advance MRA in these regions. Likewise, the JEMRA secretariat strongly encourages qualified experts with experience working in LMICs to respond to calls for data and experts, lending more contributions from various perspectives from diverse food systems.

The challenges and hazards in the food system change: new pathogens evolve, novel food processing technologies, and products become more popular. As the food system and the science evolves, methodologies need to be updated to keep pace with state of the scientific knowledge. For example, one of the most recent publication, microbiological risk assessment guidance for food (MRA 36, Table 1) provides a structured framework for assessing the risk of microbiological hazards in food. It consolidated and updated three previous MRA guidance documents, providing an overall umbrella for microbiological risk assessment. In a similar fashion, some other recent meetings were held to discuss *Listeria monocytogenes* in 2020 and to re-evaluate the microbiological hazards in fruits and vegetables in 2021 to provide up-to-date information that was previously published in MRA publications. In doing so, JEMRA captures recent growth and experience in this field, which continues to evolve in line with science and risk management demands.

We view international MRA, based on the objective evaluation of all available evidence from production to consumption, as valuable resource for Codex and others. JEMRA will continue to provide such advice as requested, as well as update advice as needed and work on issues identified as important and emerging. Continued work in this area is dependent upon national-level contributions to the cause, not only for financial resources, but also to provide all the necessary data and continued participation of their experts.

## Figures and Tables

**Table 1:** List of publications in the FAO/WHO microbiological risk assessment (MRA) series.

MRA Vol. Number	MRA Publication Title
1	Risk assessments of *Salmonella* in eggs and broiler chickens: interpretative summary
2	Risk assessments of *Salmonella* in eggs and broiler chickens
3	Hazard characterization for pathogens in food and water: guidelines
4	Risk assessment of *Listeria monocytogenes* in ready-to-eat foods: interpretative summary
5	Risk assessment of *Listeria monocytogenes* in ready-to-eat foods: technical report
6	*Enterobacter sakazakii* and other microorganisms in powdered infant formula: meeting report
7	Exposure assessment of microbiological hazards in food: guidelines
8	Risk assessment of *Vibrio vulnificus* in raw oysters: interpretative summary and technical report
9	Risk assessment of choleragenic *Vibrio cholerae* O1 and O139 in warm-water shrimp in international trade: interpretative summary and technical report
10	*Enterobacter sakazakii* and *Salmonella* in powdered infant formula: meeting report
11	Risk assessment of *Campylobacter* spp. in broiler chickens: interpretative summary
12	Risk assessment of *Campylobacter* spp. in broiler chickens: technical report
13	Viruses in food: scientific advice to support risk management activities: meeting report
14	Microbiological hazards in fresh leafy vegetables and herbs: meeting report
15	*Enterobacter sakazakii* (*Cronobacter* spp.) in powdered follow-up formula: meeting report
16	Risk assessment of *Vibrio parahaemolyticus* in seafood: interpretative summary and technical report
17	Risk characterization of microbiological hazards in food: guidelines
18	Enterohaemorrhagic *Escherichia coli* in raw beef and beef products: approaches for the provision of scientific advice
19	*Salmonella* and *Campylobacter* in chicken meat: meeting report
20	Risk assessment tools for *Vibrio parahaemolyticus* and *Vibrio vulnificus* associated with seafood: meeting report
21	*Salmonella* spp. In bivalve mollusks: risk assessment and meeting report (in press)
22	Selection and application of methods for the detection and enumeration of human pathogenic *Vibrio* spp. in seafood: guidance
23	Multicriteria-based ranking for risk management of food-borne parasites
24	Statistical aspects of microbiological criteria related to foods: a risk managers guide
25	Risk-based approach for the control of *Trichinella* spp. and *Taenia saginata* in meat: meeting report
26	Ranking of low moisture foods in support of microbiological risk management: meeting report and systematic review (in press)
27	Microbiological hazards associated with spices and dried aromatic herbs: meeting report (in press)
28	Microbial safety of lipid based ready-to-use foods for management of moderate acute malnutrition and severe acute malnutrition: first report
29	Microbial safety of lipid based ready-to-use foods for management of moderate acute malnutrition and severe acute malnutrition: second report
30	Interventions for the control of non-typhoidal *Salmonella* spp. in beef and pork: meeting report and systematic review
31	Shiga toxin-producing *Escherichia coli* (STEC) and food: attribution, characterization, and monitoring
32	Attributing illness caused by Shiga toxin-producing *Escherichia coli* (STEC) to specific foods
33	Safety and quality of water used in food production and processing
34	Foodborne antimicrobial resistance: role of the environment, crops, and biocides
35	Advances in science and risk assessment tools for *Vibrio parahaemolyicus* and *V. vulnificus* associated with seafood
36	Microbiological risk assessment guidance for food
37	Safety and quality of water used with fresh fruits and vegetables (in press)
38	*Listeria monocytogenes* in ready-to-eat foods: attribution, characterization, and monitoring (in press)
TBD	Control measures for Shiga toxin-producing *Escherichia coli* (STEC) associated with beef and dairy products (in development)
TBD	Water use and reuse in dairy production and processing (in development)
TBD	Water use and reuse in fisheries production and processing (in development)
TBD	Microbiological hazards in fresh fruits and vegetables (in development)

**Table 2:** Pathogen-commodity topics addressed by specific MRA series publication volume numbers.

	*Salmonella* spp.	*Listeria monocytogenes*	*Cronobacter (Enterobacter) Sakazakii*,	*Vibrio* spp.	*Campylobacter* spp.	*E. coli* spp.	Parasites	Viruses
Eggs	1, 2, 34							
Poultry	1, 2, 19, 34				11, 12, 19, 34			
Dairy		4, 5, 34				31, 32, 34	23	
Infant formula and lipid-based foods for children	10, 26, 28, 29, 34	26, 28, 29, 34	6, 10, 15., 26, 28, 29, 34			26, 28, 29, 34		
Fish and seafood	21, 33, 34	4, 5, 34		8, 9, 16, 20, 22, 33, 34, 35			23, 33	13, 33
Fresh fruit and vegetables	14, 33, 34, 37	14, 33, 34, 37		14, 33, 34	14, 34	31, 32, 33, 34, 37	14, 23, 33, 37	14, 13, 33, 37
Meat	30, 34	4, 5, 34				18, 31, 32, 34	25	
Spices and herbs	14, 26, 27, 34, 37	14, 26, 34, 37		26, 14, 34	14, 34	26, 34, 37	14, 37	14, 37
Low moisture foods	26, 34	26, 34	26, 34			26, 34		
Water	33, 34, 37	33, 34, 37		33, 34		33, 34, 37	33, 37	13, 33, 37

**Table 3:** International standards informed by scientific advice provided by JEMRA and captured as part of the MRA series publications.

International Standards and Codes of Practices	Year of Adoption and Amendments	MRA Publication Vol. for Contribution of Scientific Advice
CAC/GL 21-1997 Principles and Guidelines for the Establishment and Application of Microbiological Criteria Related to Foods	Revised and renamed 2013	17, 24
CAC/GL 30-1999 Principles and Guidelines for the Conduct of Microbiological Risk Assessment	Adopted 1999. Amendments 2012, 2014	3, 7,17, 18, 36
(WHO) Guidelines for Drinking-Water Quality (GDWQ)	4th edition, incorporating the 1st addendum, 2017	3
CXC 53-2003 Code of Hygienic Practice for Fresh Fruits and Vegetables	Adopted in 2003. Revised in: 2010 (new Annex III for fresh leafy vegetables), 2012 (new Annex IV for Melons), 2013 (new Annex V for Berries), 2017.	14, 37
CXC 52-2003 Code of Practice for Fish and Fishery Products	Adopted in 2003. Revised in 2004, 2005, 2007, 2008, 2010, 2011, 2016.Amended in 2011, 2013, 2016.	33
CXC/RCP 61-2005 Code of Practice to Minimize and Contain Antimicrobial Resistance	Adopted in 2005, revision in progress	34
CAC/RCP 58-2005 Code of Hygienic Practice for Meat	Adopted in 2005. Editorial amendments 2013	18
CAC/GL 61-2007 Guidelines on the Application of General Principles of Food Hygiene to the Control of *Listeria Monocytogenes* in Foods	Adopted in 2007; Annexes II and III adopted in 2009	4,5
CAC/RCP 66-2008 Code of Hygienic Practice for Powdered Formulae for Infants and Young Children	Adopted in 2008, Annex II adopted in 2009	6, 10, 15
CAC/GL 73-2010 Guidelines on the Application of General Principles of Food Hygiene to the Control of Pathogenic Vibrio Species in Seafood	2010	8, 9, 16, 20, 21, 22, 35
CAC/GL 78-2011 Guidelines for the Control of *Campylobacter* and *Salmonella* in Chicken Meat	2011	1, 2, 11, 12, 19
CAC/GL 79-2012 Guidelines on the Application of General Principles of Food Hygiene to the Control of Viruses in Food	2012	13
CAC/GL 85-2014 Guidelines for the Control of *Taenia Saginata* in Meat of Domestic Cattle	2014	25
CAC/GL 86-2015 Guidelines for the Control of *Trichinella* Spp. in Meat of Suidae	2015	25
CXC 75-2015 Code of Hygienic Practice for Low-Moisture Foods	Adopted in 2015. Revised in 2016. Amended in 2018	26, 27
CAC/GL 88-2016 Guidelines on the Application of General Principles of Food Hygiene to the Control of Foodborne Parasites	Adopted 2016	23
CAC/GL 87-2016 Guidelines for the Control of Nontyphoidal *Salmonella* spp. in Beef and Pork Meat	2016	30
Draft Guidelines For Ready To Use Therapeutic Foods (RUTF)	In progress and also for WFP UNICEF	28, 29
Draft Guidelines For The Control Of STEC In Beef, Raw Milk and Cheese Produced From Raw Milk, Leafy Greens, and Sprouts	In progress	31,32
Draft Guidelines on Integrated Monitoring and Surveillance of Foodborne Antimicrobial Resistance	In Progress	34

## Data Availability

We are happy make the raw data of the number of citations per year for the MRA documents available, but it is clearly depicted in Figure 3. The methods for data aquistion are clearly described (search on Web of Science) and repeatable.
